# Household Firearm Ownership and Firearm Mortality

**DOI:** 10.1001/jamanetworkopen.2024.29335

**Published:** 2024-08-21

**Authors:** Andrew R. Morral, Denis Agniel, Rosanna Smart, Terry L. Schell

**Affiliations:** 1 Gun Policy in America Initiative, RAND Corporation, Arlington, Virginia

## Abstract

**Importance:**

Causal associations between household firearm ownership rates (HFRs) and firearm mortality rates are not well understood.

**Objective:**

To assess the population-level temporal sequencing of firearm death rates and HFRs.

**Design, Setting, and Participants:**

This cohort study used autoregressive cross-lagged models to analyze HFRs, firearm suicide rates, and firearm homicide rates in the US from 1990 to 2018. The suicide analyses included 16 demographic subgroups of adults, defined by study year, state, sex, race and ethnicity, marital status, and urbanicity. The homicide analyses consisted of adult subgroups living in urban or rural areas. Data analysis was conducted from March to December 2023.

**Exposures:**

Firearm mortality rates and HFRs.

**Main Outcomes and Measures:**

Firearm homicide and suicide rates with HFRs as the exposure, and HFR with mortality as the exposure.

**Results:**

A total of 10 416 observations of 16 demographic subgroups by state and 2-year periods were included in the suicide analyses, while 1302 observations from 2 demographic subgroups by state and 2-year period were included in the homicide analysis. At baseline, the mean (SD) rate per 100 000 population across strata was 7.46 (7.21) for firearm suicides and 3.32 (2.13) for firearm homicides. The mean (SD) baseline HFR was 36.9% (20.2%) for firearm suicides and 36.9% (14.8%) for firearm homicides. Higher HFR preceded increases in suicide rates: demographic strata with equal firearm suicide rates but which differ by 18.6 percentage points on HFR (1 SD) would be expected to have firearm suicide rates that diverged by 0.19 (95% CI, 0.15-0.23) deaths per 100 000 population per period. With these differences accumulated over 8 years, firearm suicide rates in subgroups with the highest decile HFR would be expected to have 1.93 (95% CI, 1.64-2.36) more suicides per 100 000 population than strata with lowest decile HFR, a difference of 25.7% of the overall firearm suicide rate in 2018 and 2019. Firearm suicide rates had a smaller magnitude of association with subsequent changes in HFR: strata with equal HFRs but which differ by 1 SD in firearm suicide rates had minimal subsequent change in HFRs (−0.02 [95% CI, −0.04 to 0.01] percentage points). A 1-SD difference in HFRs was associated with little difference in next-period overall firearm homicides rates (0.03 [95% CI, −0.02 to 0.08] per 100 000 population), but a 1-SD difference in homicide rates was associated with a decrease in HFR (−0.09 [95% CI, −0.16 to −0.04] percentage points).

**Conclusions and Relevance:**

This cohort study found an association between high HFRs and subsequent increases in rates of firearm suicide. In contrast, higher firearm homicide rates preceded decreases in HFRs. By demonstrating the temporal sequencing of firearm ownership and mortality, this study may help to rule out some theories of why gun ownership and firearm mortality are associated at the population level.

## Key Points

**Question:**

Do population-level household firearm ownership rates (HFRs) precede changes in firearm mortality rates, do mortality rates precede changes in HFRs, or both?

**Findings:**

In this cohort study involving 16 demographic subgroups across the US, higher HFRs preceded increases in firearm suicide rates but were not associated with subsequent changes in firearm homicide rates. In contrast, higher firearm suicide rates were not associated with subsequent changes in HFRs, although higher firearm homicide rates preceded reductions in HFRs.

**Meaning:**

By demonstrating the temporal sequencing of firearm ownership and mortality, this study may help to rule out some theories of why gun ownership and firearm mortality are associated at the population level.

## Teaser

This cohort study evaluates the population-level temporal sequencing of firearm death rates and household firearm ownership rates among US adults from 1990 to 2018.

## Introduction

Firearm homicide and suicide are associated with household firearm ownership at the population and individual levels.^1,2,3,4,5,6^ However, the mechanisms underlying these associations are not well understood, limiting the literature’s usefulness for guiding public health and policy. If individuals who are already at a high risk of dying from firearm injuries are more likely to acquire firearms, then firearm ownership itself may not confer additional mortality risk. Isolating the potentially reciprocal causal association between firearm ownership and firearm mortality is methodologically challenging, however. In this cohort study, we investigated the timing of changes in firearm ownership and firearm mortality to ascertain if 1 variable preceded subsequent changes to the other, thereby establishing a necessary but not sufficient condition for a causal association between firearm ownership and mortality.

Existing evidence suggests that firearm ownership may increase suicide risk but also that people planning a suicide may be more likely to acquire a firearm. The risk of suicide is greatest immediately after acquiring a firearm, suggesting that gun acquisition may be part of a suicide plan, but elevated risk persists even years after the initial acquisition.^4,7^ Firearms are more lethal than most other suicide means,^8^ suicide attempts are often impulsive, and people who survive even serious suicide attempts rarely die by suicide at a later time.^9^ Therefore, access to and use of more lethal means may result in higher suicide rates and fewer survivors who might otherwise not die by suicide.

With few exceptions,^10^ firearm ownership at the city, county, or state level has been shown to be associated with total and firearm homicide rates, although not with nonfirearm homicide rates.^2,6,11,12,13^ Additionally, non–gun owners living with someone who acquires a firearm die by homicide at twice the rate as similar people who do not live with someone who acquires a firearm; here too, the extra risk is primarily attributed to elevated firearm-specific homicide risk.^5^ The mechanisms underlying these associations are unclear. High or increasing firearm violence rates might affect people’s decision to acquire firearms for self-protection.^14,15^ Alternatively, high rates of firearm ownership may increase diversion of legal firearms to illegal gun markets, thus increasing firearm crime. Similarly, the easy availability of firearms may embolden criminals or transform less violent encounters into homicides.^3^

For both suicides and homicides, theories point to potential bidirectional relationships between firearm ownership and violence: firearm ownership increasing firearm suicide and violence, and interpersonal violence and suicidality increasing firearm ownership.

In this study, we aimed to assess the population-level temporal sequencing of firearm death rates and household firearm ownership rates (HFRs), defined as the proportion of adults living in a household in which 1 or more firearms are kept. We used estimates of HFR for 16 population subgroups in each state from 1990 to 2018. We estimated cross-lagged models separately for firearm suicides and firearm homicides. This approach allowed us to evaluate whether HFR preceded subsequent changes in firearm mortality and whether firearm mortality preceded subsequent changes in HFR within population subgroups over the 29-year study period. To our knowledge, this study was the first to use detailed subpopulation and substate estimates of HFR to assess the potentially reciprocal temporal precedence between population-level HFR and firearm mortality.

## Methods

The RAND Institutional Review Board approved the study and waived the informed consent requirement because deidentified population data were used. Analytic methods for the firearm homicide analyses were preregistered at OSF Registries. We followed the Strengthening the Reporting of Observational Studies in Epidemiology (STROBE) reporting guideline.

### Data Sources

#### HFRs

Morral et al^16^ provided estimates of HFR by year (from 1990 to 2018), US state, and 16 demographic subgroups, defined by the cross-classification of 4 dichotomous indicators: race and ethnicity (White [including Alaskan Native, American Indian, and non-Hispanic White] vs other [including Asian, Black, other Hispanic, and other single racial and ethnic groups]), sex (male vs female), marital status (married vs unmarried), and county urbanicity (urban vs nonurban). Race and ethnicity data were collected and analyzed because of known large racial disparities in exposure to both firearm homicide and firearm suicide. Applying small-area estimation techniques, a machine-learning model was used to evaluate self-reported household gun ownership from over 700 000 survey respondents (from 16 administrations of the General Social Survey^17^ and 3 administrations of the Behavioral Risk Factor Surveillance System^18^) as a function of state; year; sex; race and ethnicity; marital status; urbanicity; and model-based estimates of the proportion of suicides that are firearm suicides in each demographic stratum, state, and year.

#### Mortality

Firearm homicides and suicides from 1990 to 2018 came from the restricted-use, state- and all counties–level Multiple Cause of Death dataset from the National Vital Statistics System,^19^ which included information on the year, state, and county of death occurrence; mechanism of death (eg, firearm or nonfirearm); cause of death (eg, suicide or homicide); and decedent demographic information.

#### Population

Population estimates for demographic subgroups in each state and year were derived from the National Cancer Institute Surveillance, Epidemiology and End Results (SEER) Program population model.^20^ SEER estimates are constructed from the US Census Bureau county population estimates by age, sex, race, and Hispanic ethnicity. Because SEER data do not include marital status, strata defined by year, state, sex, race and ethnicity, and urbanicity were divided into married vs unmarried populations using fractions derived from the decennial Census and the American Community Survey data.^16,21^

### Statistical Analysis

Data analysis was conducted from March to December 2023. Two-sided *P* < .05 indicated statistical significance. Data analysis was performed with R, version 4.2.3 (R Project for Statistical Computing).

#### Construction of Rates by Analytic Strata

Each subgroup’s annual rates were combined into 15 two-year periods (1990 and 1991 through 2018 and 2019) because the HFR measure was derived from data available every other year. Mortality rates were expressed per 100 000 of the relevant population.

For suicides, all 16 demographic subgroups in each state and 2-year period served as units of analysis. For firearm homicides, 2 demographic subgroups were constructed: urban and rural for every state-period. We used this different aggregation for homicides because, unlike with suicides, the demographic characteristics of the perpetrators in firearm homicides are often unknown and may differ from those of the decedents. To better align the characteristics of the decedents with the HFR of the perpetrators, we differentiated only between urban vs nonurban residency, because most people who die by homicide are killed by people who live near them.^22^

#### Cross-Lagged Models

We estimated separate cross-lagged models of firearm mortality rates and HFR for homicide and suicide outcomes. In each model, each variable was regressed on the prior period’s HFR and mortality rate. The models were stationary; that is, the autoregressive regression coefficient, the cross-lagged regression coefficient, and the error variances were time invariant. These models also included time fixed effects on each outcome to account for national patterns. In addition to analyzing the coefficients from these 2 models, we computed the covariance between the residuals of the 2 models to assess whether contemporaneous changes in ownership and mortality (unexplained by the cross-lags) were associated. Both models were fit using weighted least squares, where weights corresponded to the subgroup population during the period. We quantified cumulative magnitudes of the estimated associations across multiple periods by fixing ownership and mortality rates in 2010 and 2011 and estimating the marginal effects 8 years later (2018 and 2019). This approach accounted for the reciprocal associations between the 2 constructs and the stability of each construct over time.

We estimated SEs and *P* values by resampling strata-years 1000 times, rerunning the model for each to establish bootstrap distributions.^23,24^ The bootstrap allowed us (1) to estimate the SEs of model coefficients without assuming the model was correct or the normality of errors and (2) to estimate SEs for functions of both models (eg, for cumulative associations).

We repeated these models on subgroups stratified by sex and race and ethnicity (as well as urbanicity for the homicide analyses). As a sensitivity analysis, we ran models for nonfirearm suicides and nonfirearm homicides.

## Results

The suicide analyses included 10 416 observations from 16 demographic subgroups per state per period. The homicide analyses consisted of 1302 observations from urban and rural demographic subgroups per state per period. At baseline, the mean (SD) rate per 100 000 population across strata was 7.46 (7.21) for firearm suicide and 3.32 (2.13) for firearm homicide. Mean (SD) HFR at baseline was 36.9% (20.2%) for firearm suicides and 36.9% (14.8%) for firearm homicides.

**Figure 1.**
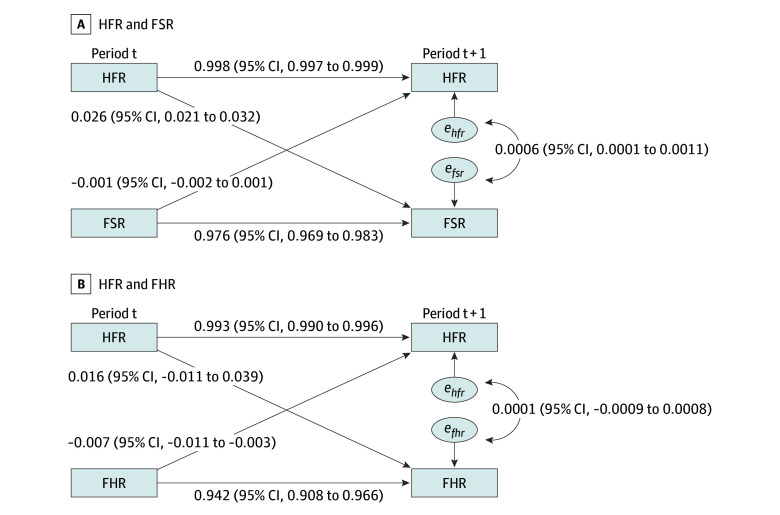
Standardized Coefficients for Cross-Lagged Models of Household Firearm Ownership Rate (HFR) and Firearm Mortality FHR indicates firearm homicide rate; FSR, firearm suicide rate. The *e_hfr_, e_fsr_, e_fhr_* are model residual terms. The full model includes period fixed effects.

While there was substantial variation across strata in HFRs, firearm homicide rates, and firearm suicide rates (Table 1 and Table 2; eFigures 1 and 2 in Supplement 1), the cross-lagged model results demonstrated little variation in these measures within strata over time. The autoregressive coefficients estimated by the models all exceeded 0.94 (Figure 1), indicating that all 3 measures evolved slowly over time. With such stable time series, we expected cross-lagged associations to be small in absolute magnitude even if they were substantively associated with changes from 1 period to the next.

**Table 1.** Subgroup Characteristics in Firearm Suicide Analyses Averaged Across US States and 2-Year Periods From 1990 and 1991 Through 2018 and 2019

Sex	Race and ethnicity^a^	Marital status	Area of residence	Mean population size, No.	Mean (SD)	
					Firearm suicide rate, No.	HFR, %^b^
Male	White	Unmarried	Rural	477 032	18.8 (6.5)	52.7 (12.4)
Male	White	Unmarried	Urban	678 039	16.2 (8.9)	35.7 (10.8)
Male	White	Married	Rural	424 847	15.8 (5.4)	62.8 (14.0)
Male	White	Married	Urban	566 224	12.8 (5.8)	44.0 (13.2)
Male	Other	Married	Rural	68 290	6.2 (5.4)	42.3 (13.3)
Male	Other	Unmarried	Rural	148 598	5.9 (4.7)	35.0 (11.1)
Male	Other	Unmarried	Urban	527 638	5.8 (3.8)	23.5 (9.3)
Male	Other	Married	Urban	261 465	5.3 (3.8)	26.1 (10.3)
Female	White	Married	Rural	419 682	2.6 (1.5)	54.7 (13.9)
Female	White	Unmarried	Urban	743 969	2.5 (2.0)	15.1 (6.9)
Female	White	Unmarried	Rural	509 296	2.5 (1.5)	24.6 (8.9)
Female	White	Married	Urban	556 051	2.3 (1.9)	35.6 (12.1)
Female	Other	Married	Rural	63 239	0.8 (1.7)	32.6 (11.9)
Female	Other	Unmarried	Urban	576 000	0.7 (1.0)	9.6 (4.8)
Female	Other	Married	Urban	254 308	0.7 (1.0)	18.8 (8.2)
Female	Other	Unmarried	Rural	149 277	0.7 (1.2)	15.0 (6.4)

Abbreviation: HFR, household firearm ownership rate.

^a^ Race and ethnicity are dichotomized as White (including Alaskan Native, American Indian, and non-Hispanic White) or other (including Asian, Black, other Hispanic, and other single race or ethnicity) races and ethnicities.

^b^ HFR is the proportion of adults living in a household in which 1 or more firearms are kept.

**Table 2.** Subgroup Characteristics in Firearm Homicide Analyses Averaged Across US States and 2-Year Periods From 1990 and 1991 Through 2018 and 2019

Area of residence	Mean population size, No.	Mean (SD)	
		Firearm homicide rate, No.	HFR, %^a^
Rural	4 355 257	2.4 (1.7)	44.6 (11.6)
Urban	8 014 157	5.9 (5.0)	27.2 (9.5)

Abbreviation: HFR, household firearm ownership rate.

^a^ HFR is the proportion of adults living in a household in which 1 or more firearms are kept.

The residual covariances with a positive value (Figure 1) indicated that, accounting for cross-lagged associations, increases in both firearm suicide and firearm homicide rates were associated with contemporaneous increases in HFRs. However, these associations and their 95% CIs included only estimates that were small in magnitude.

### Suicide Rate and HFR

**Figure 2.**
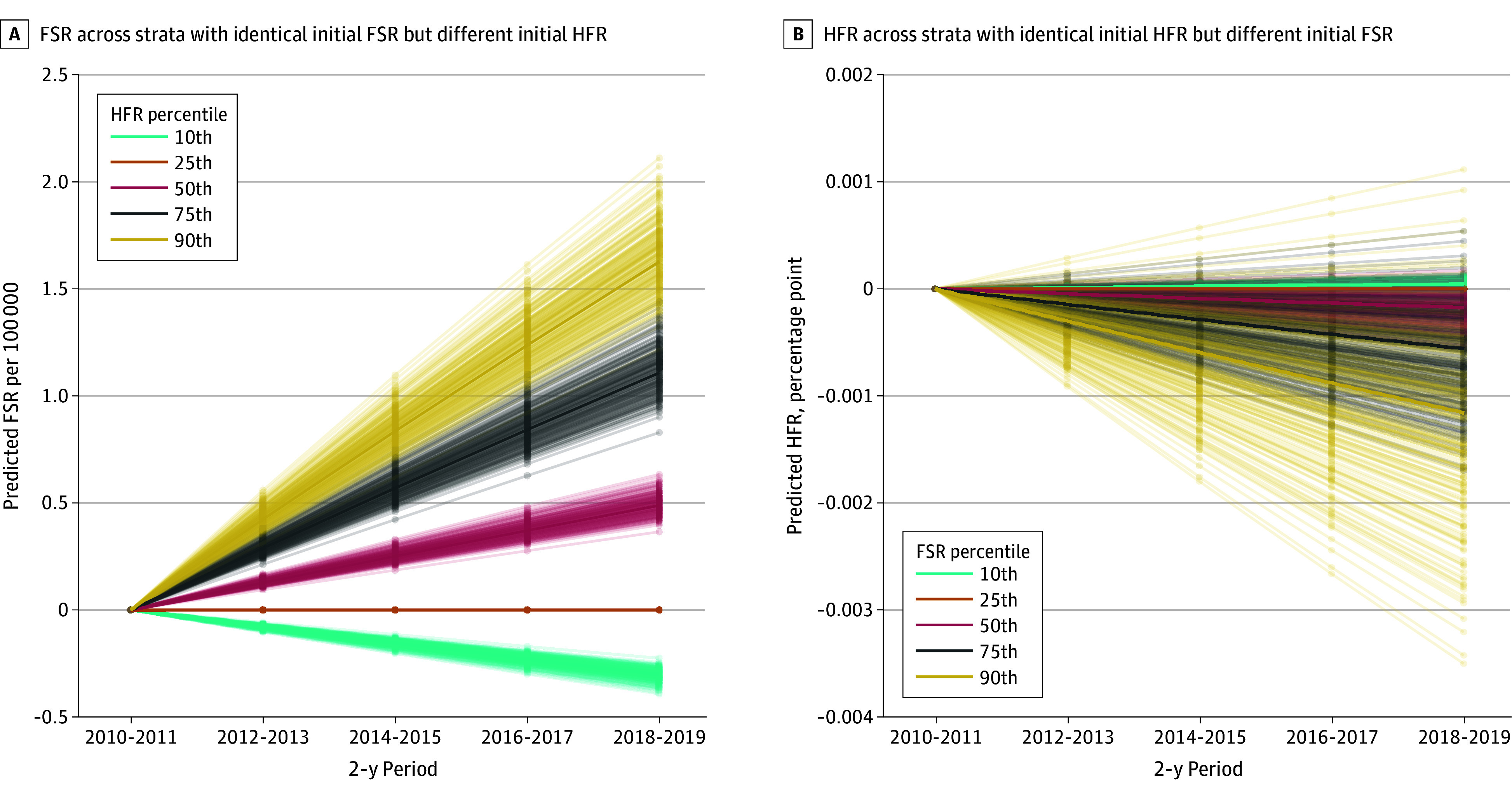
Extrapolations of Cross-Lagged Household Firearm Ownership Rate (HFR) and Firearm Suicide Rate (FSR) Outcomes Over 8 Years Ending in 2019 Differences in expected FSR and HFR are expressed relative to the expected rate for the 25th percentile FSR or HFR. The ordinate scale (y-axis) spans 0.34 of an SD in FSR (A) and .02 of an SD in HFR (B).

The cross-lagged coefficient from HFRs to subsequent suicide rates (0.026; 95% CI, 0.021-0.032; *P* < .001) was positive and significant, whereas the coefficient for the association between suicide rates and subsequent HFRs was not significant (Figure 1A; eTable 1 in Supplement 1 shows standardized coefficients for these associations). The standardized cross-lagged coefficient of the association between HFRs and suicide rates (0.026) implied that a subgroup with an HFR 18.6 percentage points higher than the HFR of another subgroup but with the same suicide rates at 1 time point would be expected to have 0.19 (95% CI, 0.15-0.23) more suicides per 100 000 population in the next 2-year period than a subgroup with a lower HFR. These associations represent the expected change from one 2-year period to the next. However, such associations accumulate when looking at expectations over longer periods and are more relevant for variables such as HFRs and firearm suicide rates, which change slowly. Figure 2A illustrates the differences in expected firearm suicide rates over time for subgroups with the same initial firearm suicide rates but different initial HFRs (corresponding to the 10th, 25th, 50th, 75th, and 90th percentiles of HFR nationally). After 8 years, firearm suicide rates between the highest and lowest HFR deciles would be expected to diverge by 1.93 (95% CI, 1.64-2.36) suicides per 100 000 population or by 25.7% of the US firearm suicide rate in 2018 and 2019.

In contrast, the estimated magnitude of the association between firearm suicide rate and subsequent HFR was small in the primary specification and in all sensitivity tests (eTable 1 in Supplement 1). We estimated that 2 subgroups with the same current-year HFRs but differing by 1 SD in firearm suicide rates (7.34 per 100 000 population) would experience changes in HFR that differed by only −0.02 (95% CI, −0.04 to 0.01) percentage points in the next 2-year period (less than 2 one-hundredths of an SD). With these associations aggregated over time, 2 strata with equal initial HFRs but substantially different firearm suicide rates (90th vs 10th percentile) would be expected to differ in HFR by 0.1 percentage points 8 years later (Figure 2B).

### Homicide Rate and HFR

**Figure 3.**
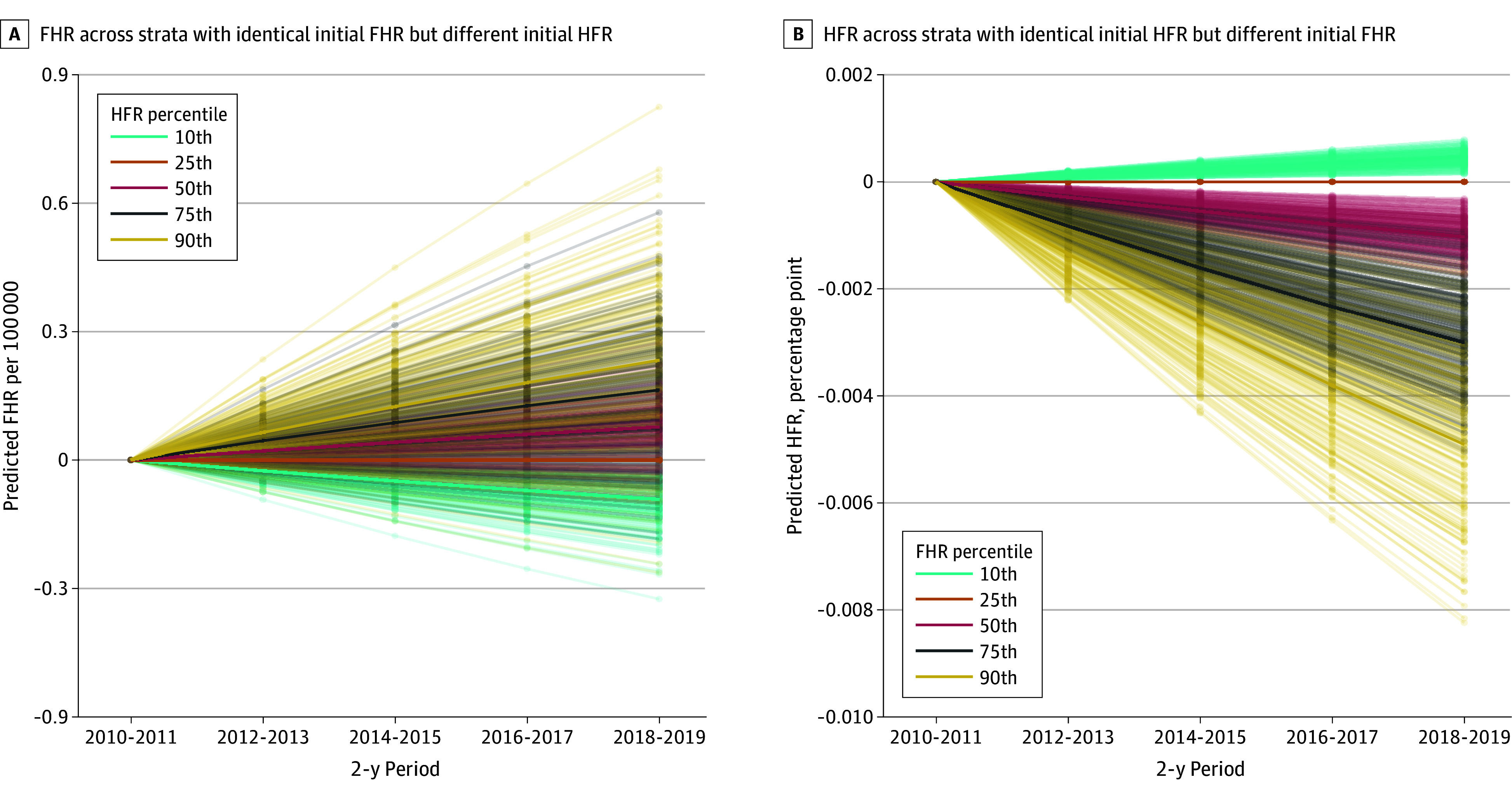
Extrapolations of Cross-Lagged Household Firearm Ownership Rate (HFR) and Firearm Homicide Rate (FHR) Outcomes Over 8 Years Ending in 2019 Differences in expected FHR and HFR are expressed relative to the expected rate for the 25th percentile FHR or HFR. The ordinate scale (y-axis) spans 0.45 of an SD in FHR (A) and .06 of an SD in HFR (B).

We did not find significant evidence that subgroups with higher HFRs, on average, had larger increases in firearm homicide rates in the next 2-year period (Figure 1B). Specifically, we estimated that the next-period homicide rates in 2 subgroups with the same current-period homicide rates but differing by 1 SD in HFRs (13.8 percentage points) would differ by 0.03 (95% CI, −0.02 to 0.08) homicides per 100 000 population (0.02 of an SD). These associations accumulated over time (Figure 3A) implied that after 8 years, firearm homicide rates between groups that were initially in the highest and lowest HFR deciles would be expected to diverge by 0.33 (95% CI, −0.20 to 0.78) homicides per 100 000 population or by 7.7% of the US firearm homicide rate in 2018 and 2019.

In contrast, higher firearm homicide rates preceded small reductions in HFRs over time. Two strata with the same current-year HFRs but differing by 1 SD in the firearm homicide rates (2.02 per 100 000 population) would be expected to have their HFRs diverge by −0.09 (95% CI, −0.16 to −0.04) percentage points in the next 2-year period (approximately one-tenth of a percentage point or 0.01 of an SD). However, even after accumulating associations over an 8-year period, strata with equal initial HFRs but substantially different firearm homicide rates (90th vs 10th percentile) would be expected to have their HFRs diverge by only 0.54 percentage points or 0.04 of an SD (Figure 3B).

### Subgroup and Sensitivity Tests

As in the primary analyses, all subgroup tests found that higher HFRs were associated with next-period increases in firearm suicide rates, although these associations were greater in magnitude for males (0.26 [95% CI, 0.20-.36] more suicides per 100 000 population) and populations with other race and ethnicity (0.36; 95% CI, 0.27-.49) than for females (0.08; 95% CI, 0.05-.12) and White populations (0.09; 95% CI, 0.05-.13) (eTables 1 and 2 in Supplement 1). For males, this relative increase in firearm suicides was equivalent to an additional 4.08 suicides per 100 000 population over 8 years. Higher firearm suicide rates had small increases in HFRs for males (0.0007; 95% CI, 0.0004-.0011) but decreases for White populations (–0.003; 95% CI, –0.0005 to –0.0001), with negligible associations for populations with other race and ethnicity. Sensitivity analyses using nonfirearm suicide rates found that higher HFRs were associated with small but significant increases in nonfirearm suicide rates (0.07 [95% CI, 0.05-.10] additional deaths per 100 000 population). This association of HFR and subsequent nonfirearm suicide rates corresponds to approximately 40% of the magnitude of HFR association with firearm suicide rates.

As in the primary analyses, a 1-SD difference in HFRs preceded small and uncertain changes in next-period firearm homicide rates in most analyses stratified by race and ethnicity, sex, and urbanicity (eTables 3 and 4 in Supplement 1). However, we found that higher HFR preceded increases in next-period firearm homicide rates among females (0.01 [95% CI, 0.00-.01] additional deaths per 100 000 population) and White individuals (0.02; 95% CI, 0.00-.04). These associations were small in magnitude and corresponded to less than 1% of firearm homicide rates for these groups in 2018 and 2019. Similar to the primary analyses, all subgroup analyses showed that higher initial firearm homicide rates were consistently associated with small reductions in HFRs (eTables 3 to 5 in Supplement 1). Models including nonfirearm homicide rates also showed that higher nonfirearm homicide rates were associated with later decreases in HFR (–0.001; 95% CI, –0.002 to –0.000) that were comparable to those found with increases in firearm homicide rates.

## Discussion

To our knowledge, this study provides the first longitudinal evidence describing the temporal sequencing of HFRs and firearm mortality rates for state and substate population groups. We found consistent evidence that groups with higher HFRs have relative increases in firearm suicides over time, but there was little evidence that higher firearm suicide rates have a reciprocal association with subsequent HFRs. This finding was more pronounced in males, for whom a 1-SD difference in HFRs was associated with relatively large differences in firearm suicide rates over an 8-year period (eg, 4.08 suicides per 100 000 population, almost a third of the 2018 and 2019 firearm suicide rates for males).

In the primary models, HFRs were not associated with changes in firearm homicide rates. These nonsignificant results may partially reflect the considerably wider 95% CIs for cross-lags estimating firearm homicide rates compared with the analogous 95% CIs in the firearm suicide rate models. The 95% CIs for the association between HFRs and subsequent homicide rates did not rule out the possibility of meaningfully large associations. In addition, results of the subgroup tests by sex and race and ethnicity suggested that high HFRs may precede increased homicides in female and White populations. Although prior research found a contemporaneous association between HFRs and homicides in females,^11^ the present study’s finding of a temporal sequencing of changes in HFRs and subsequent firearm homicide rates, although small in magnitude, adds new evidence for a possible, but not yet proven, role of firearm access in femicides. Intimate partners are the perpetrators in more than a third of all homicides in females compared with 6% of homicides in males,^25^ which may help explain why the association of HFRs and homicide rates was more evident for females.

High firearm homicide rates reliably preceded greater reduction in HFRs, compared with lower homicide rates. This finding contradicts the suggestion that homicides and HFRs are related because people arm themselves as a response to high firearm homicide rates.^15^ This could be true for some individuals, but at the population level higher firearm homicide rates precede decreases in HFRs. The mechanisms underlying this association require future research, although this association may be attributed to social processes, such as stigma of firearms among groups burdened with high firearm violence, perceptions of risk of owning a firearm, outmigration of populations with high HFR from high-violence to low-violence counties, or policy interventions in response to violence that make acquiring or keeping a firearm more difficult.

These findings should not be interpreted as demonstrating a causal association between HFRs and subsequent suicide rates or between homicide rates and subsequent HFRs. Although we found reliable temporal sequencing between these changes, this order is a necessary but not sufficient condition for demonstrating causal associations. Some of the temporal ordering we observed could be due to other factors affecting both HFRs and mortality rates, such as legal and policy interventions, economic conditions, and mass media. For example, the finding that HFRs preceded subsequent firearm and nonfirearm suicides may reflect a causal association of HFR with both outcomes (eg, through direct and contagion events) or may suggest that broader changes in social norms or conditions are affecting all 3 outcomes but with differential lags. Thus, while we do not interpret as causal the longitudinal association of higher firearm homicide rates and subsequent reductions in HFSs, this interpretation is inconsistent with theories that population-level gun ownership rates increase in response to high homicide rates.^15^ Similarly, while we cannot conclude that high HFRs cause the increased suicide rates that follow, the findings support such a theory. Furthermore, the finding that HFRs are associated with increasing suicide rates should be considered by both the public and policymakers when making personal decisions and public policies regarding firearm acquisition or access.

### Limitations

This study has several limitations. First, the study was built on model-based estimates of HFR, which may imperfectly describe household firearm ownership if, for instance, the survey responses on which the model was based were inaccurate. Similarly, measurement error in both HFRs and mortality rates necessarily biased the estimates toward 0; thus, the estimated associations may be smaller than the associations disattenuated for measurement error. Second, HFR was only 1 aspect of firearm availability or exposure, and the results do not inform the relationship between firearm deaths and other possible measures (eg, public carrying, illegal market availability, density of firearm dealers, or number of firearms) that may play important roles in firearm homicides and community violence.^26,27^ Third, while this study identified associations at the population level, associations at the individual level may differ.

## Conclusions

This study found an association between high HFR and subsequent increases in suicide rates, which supports but does not confirm the role of firearm ownership in increasing the risk of suicide death. High firearm homicide rates were associated with subsequent reductions in HFRs, which is inconsistent with the theory that firearms are acquired in response to increasing violent crimes. To our knowledge, this study was the first to demonstrate an association between high HFRs and subsequent increases in firearm suicide rates across populations with different demographic and geographic characteristics. This information may help groups and individuals make informed decisions about firearm ownership.

## Notes

Supplement 1.
eMethods
**eFigure 1.** Firearm Suicide Rate Versus HFR With Loess Fit, All Analytic Strata and Periods (N = 10,416) **eFigure 2.** Firearm Homicide Rate Versus HFR With Loess Fit, All Analytic Strata and Periods (N = 1,302) **eTable 1.** Standardized Coefficients for Firearm Suicide Models **eTable 2.** Standardized Coefficients for Suicide Rate Models: Subgroup and Sensitivity Analyses **eTable 3.** Standardized Coefficients for Firearm Homicide Models **eTable 4.** Standardized Coefficients for Homicide Rate Models: Subgroup Analyses **eTable 5.** Standardized Coefficients for Homicide Rate Models: Sensitivity Analyses

Supplement 2.
Data Sharing Statement
