# Supplementary material for: Household Firearm Ownership and Firearm Mortality
Source: JAMA Netw Open. 2024 Aug 21;7(8):e2429335. doi: 10.1001/jamanetworkopen.2024.29335 (PMC11339659; doi:10.1001/jamanetworkopen.2024.29335)
Supplement: Supplement 1. — eMethods eFigure 1. Firearm Suicide Rate Versus HFR With Loess Fit, All Analytic Strata and Periods (N = 10,416) eFigure 2. Firearm Homicide Rate Versus HFR With Loess Fit, All Analytic Strata and Periods (N = 1,302) eTable 1. Standardized Coefficients for Firearm Suicide Models eTable 2. Standardized Coefficients for Suicide Rate Models: Subgroup and Sensitivity Analyses eTable 3. Standardized Coefficients for Firearm Homicide Models eTable 4. Standardized Coefficients for Homicide Rate Models: Subgroup Analyses eTable 5. Standardized Coefficients for Homicide Rate Models: Sensitivity Analyses [file jamanetwopen-e2429335-s001.pdf]

Supplemental Online Content

Morral AR, Agniel D, Smart R, Schell TL. Household firearm ownership and firearm mortality. *JAMA Netw Open*. 2024;7(8):e2429335.
doi:10.1001/jamanetworkopen.2024.29335

eMethods

eFigure 1. Firearm Suicide Rate Versus HFR With Loess Fit, All Analytic Strata and Periods (N= 10,416)

eFigure 2. Firearm Homicide Rate Versus HFR With Loess Fit, All Analytic Strata and Periods (N= 1,302)

eTable 1. Standardized Coefficients for Firearm Suicide Models

eTable 2. Standardized Coefficients for Suicide Rate Models: Subgroup and Sensitivity Analyses

eTable 3. Standardized Coefficients for Firearm Homicide Models

eTable 4. Standardized Coefficients for Homicide Rate Models: Subgroup Analyses

eTable 5. Standardized Coefficients for Homicide Rate Models: Sensitivity Analyses

This supplemental material has been provided by the authors to give readers additional information about their work.

© 2024 Morral AR et al. *JAMA Network Open*

eMethods

1. Model specification

Let Y_{it}, Z_{it} be the standardized firearm suicide rate per 100,000 and HFR, respectively, for stratum i in two-year period t. The cross-lag model is defined by the following two equations:

$$E(Y_{it}|Y_{it-1}, Z_{it-1}) = \alpha_t + \beta_1 Y_{it-1} + \beta_2 Z_{it-1}$$

where α_t denotes the time fixed effect to capture national trends in firearm suicide rate, β_1 denotes the auto-regressive term relating previous suicide rates to current suicide rates, and β_2 denotes the relationship between previous HFR and current suicide rate; and

$$E(Z_{it}|Y_{it-1}, Z_{it-1}) = \gamma_t + \zeta_1 Z_{it-1} + \zeta_2 Y_{it-1}$$

where γ_t denotes the time fixed effect to capture national trends in HFR, ζ_1 denotes the auto-regressive term relating previous HFR to current HFR, and ζ_2 denotes the relationship between previous suicide rate and current HFR.

Specification of models for other outcomes like firearm homicide rate take the same form. Weighted least squares was used to estimate coefficients from these models, where weights correspond to the population in stratum i in two-year period t. Add new final sentence to this section. Homicide rate models were preregistered at: https://osf.io/ncwds

2. Extrapolating model predictions over time

In Figures 2 and 3 in the main text, we play out the implications of the model over five two-year periods. Taking Figure 2 Panel A as an example, we fixed the suicide rate in 2010/2011 at its median value (call it y_0), and we computed the 10th, 25th, 50th, 75th, and 90th quantiles of HFR in 2010/2011. For each of the HFR quantile values (call it z_0), we predicted the 2012/2013 firearm suicide rate and HFR from the baseline values of suicide rate and HFR based on the model coefficients as

$$\widehat{\mu_1} = \widehat{\alpha_1} + \widehat{\beta_1} y_0 + \widehat{\beta_2} z_0$$
$$\widehat{m_1} = \widehat{\gamma_1} + \widehat{\zeta_1} z_0 + \widehat{\zeta_2} y_0$$

These predicted 2012/2013 values are then used to predict the 2014/2015 values again using the model coefficients as:

$$\widehat{\mu_2} = \widehat{\alpha_2} + \widehat{\beta_1}\widehat{\mu_1} + \widehat{\beta_2}\widehat{m_1}$$
$$\widehat{m_2} = \widehat{\gamma_2} + \widehat{\zeta_1}\widehat{m_1} + \widehat{\zeta_2}\widehat{\mu_1}$$

And so on for future time periods.

eFigure 1. Firearm Suicide Rate Versus HFR With Loess Fit, All Analytic Strata and Periods (N= 10,416)

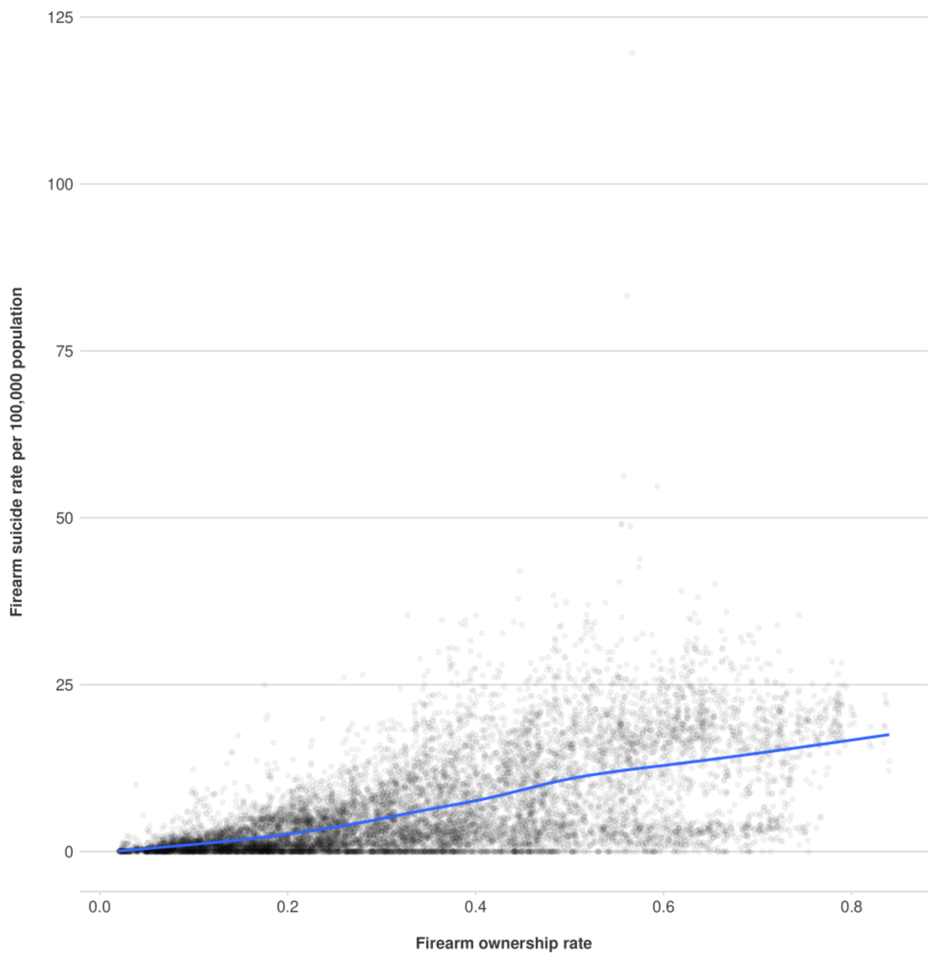

© 2024 Morral AR et al. *JAMA Network Open*

eFigure 2. Firearm Homicide Rate Versus HFR With Loess Fit, All Analytic Strata and Periods (N= 1,302)

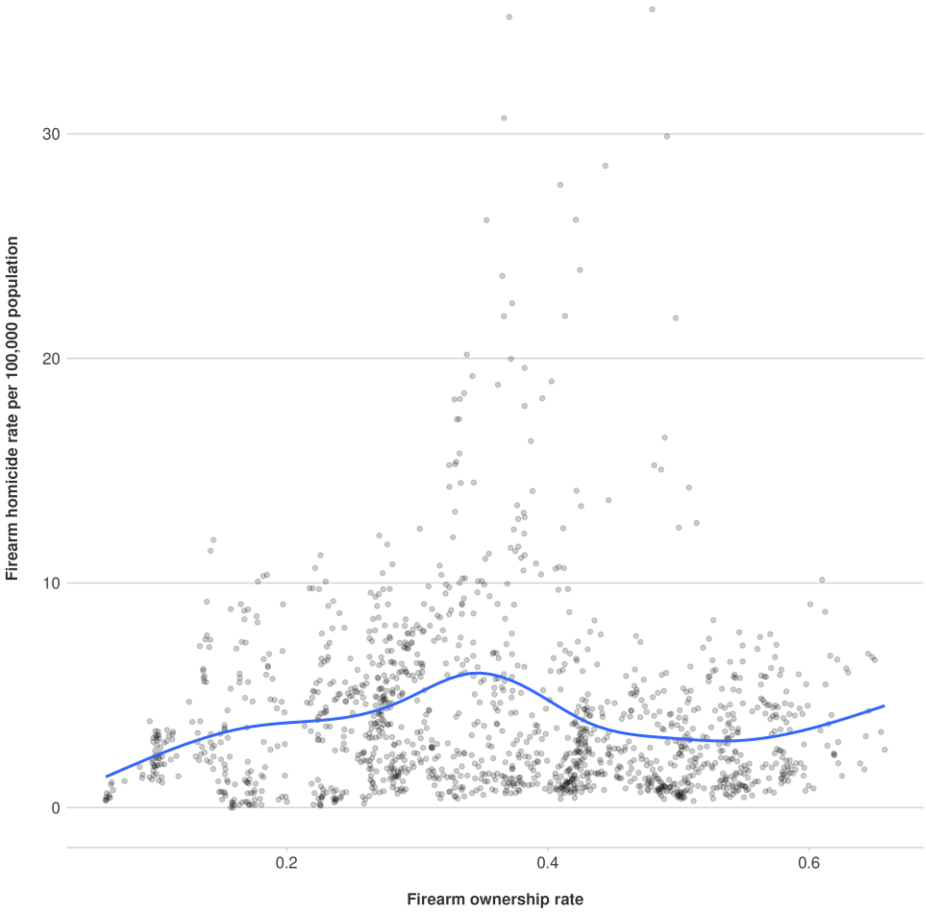

© 2024 Morral AR et al. *JAMA Network Open*

eTable 1. Standardized Coefficients for Firearm Suicide Models

Analysis	Variable	Coefficient	95% confidence interval		p
			Lower	Upper	
Full population: Main model					
	Predicting FSR from prior:				
	HFR	0.0259	0.0210	0.0315	<0.001
	FSR	0.9758	0.9686	0.9826	<0.001
	Predicting HFR from prior:				
	HFR	0.9981	0.9966	0.9994	<0.001
	FSR	-0.0008	-0.0021	0.0005	0.192
	Covariance of HFR and FSR model residuals	0.0006	0.0001	0.0011	0.027
Male victims					
	Predicting FSR from prior:				
	HFR	0.0559	0.0432	0.0732	<0.001
	FSR	0.9359	0.9144	0.9526	<0.001
	Predicting HFR from prior:				
	HFR	0.9971	0.9947	0.9994	<0.001
	FSR	0.0034	0.0009	0.0062	0.014
	Covariance of HFR and FSR model residuals	0.0009	0.0001	0.0020	0.059
Female victims					
	Predicting FSR from prior:				
	HFR	0.0825	0.066	0.1079	<0.001
	FSR	0.8478	0.8141	0.8717	<0.001
	Predicting HFR from prior:				
	HFR	0.9979	0.9961	0.9994	<0.001
	FSR	0.0005	-0.001	0.0023	0.523
	Covariance of HFR and FSR model residuals	0.0013	0.0002	0.0028	0.056

NOTE: FSR is firearm suicide rate; HFR is household firearm ownership rate. Coefficients for cross-lagged effects (HFR -> FSR; FSR -> HFR) describe the standardized expected differences in the outcome at time 2 for groups with identical outcomes at time 1 but which differ by one standard deviation on the predictor. Coefficients for the association of HFR with later HFR and FSR with later FSR are autocorrelations. The covariance of residuals are the partial correlations between these standardized variables at time *t* conditioned on their *t*-1 values.

© 2024 Morral AR et al. *JAMA Network Open*

eTable 2. Standardized Coefficients for Suicide Rate Models: Subgroup and Sensitivity Analyses

Analysis	Variable	Coefficient	95% confidence interval Lower	Upper	p
White populations: Firearm Suicide Rate (FSR)					
	Predicting FSR from prior:				
	HFR	0.0171	0.0129	0.0224	<0.001
	FSR	0.9837	0.9769	0.9892	<0.001
	Predicting HFR from prior:				
	HFR	0.9979	0.9966	0.9992	<0.001
	FSR	-0.0021	-0.0034	-0.0006	0.003
	Covariance of HFR and FSR model residuals	0.0001	-0.0003	0.0007	0.683
Nonwhite populations: Firearm Suicide Rate (FSR)					
	Predicting FSR from prior:				
	HFR	0.1183	0.0987	0.1463	<0.001
	FSR	0.7871	0.7436	0.8183	<0.001
	Predicting HFR from prior:				
	HFR	0.9862	0.983	0.9889	<0.001
	FSR	-0.0037	-0.0066	-0.0006	0.014
	Covariance of HFR and NFSR model residuals	0.001	-0.0009	0.0032	0.338
Full population: Nonfirearm Suicide Rate (NFSR)					
	Predicting NFSR from prior:				
	HFR	0.0157	0.0112	0.0220	<0.001
	NFSR	0.9769	0.9623	0.9868	<0.001
	Predicting HFR from prior:				
	HFR	0.9973	0.9961	0.9984	<0.001
	NFSR	0.0012	0.0003	0.0021	0.015
	Covariance of HFR and NFSR model residuals	0.0003	-0.0004	0.0009	0.418
Full population: Total Firearm Suicide Rate (TFSR)					
	Predicting TFS from prior:				
	HFR	0.0142	0.0110	0.0180	<0.001
	TFSR	0.9962	0.9903	1.0019	<0.001
	Predicting HFR from prior:				
	HFR	0.9975	0.9962	0.9987	<0.001
	TFSR	0.0001	-0.0010	0.0012	0.917
	Covariance of HFR and *TFSR* model residuals	0.0004	0.0000	0.0010	0.084

NOTE: HFR is household firearm ownership rate. TFSR combines firearm and nonfirearm suicides. Coefficients for cross-lagged effects (HFR -> NFSR; NFSR -> HFR) describe the standardized expected differences in the outcome at

© 2024 Morral AR et al. *JAMA Network Open*

time 2 for groups with identical outcomes at time 1 but which differ by one standard deviation on the predictor. Coefficients for the association of HFR with later HFR and NFSR with later NFSR are autocorrelations. Total firearm suicides was not discussed in the paper, but is included here for completeness. The covariance of residuals are the partial correlations between these standardized variables at time *t* conditioned on their *t*-1 values.

eTable 3. Standardized Coefficients for Firearm Homicide Models

Analysis	Variable	Coefficient	95% confidence interval Lower	Upper	p
Full population: Main model					
	Predicting FHR from prior:				
	HFR	0.0164	-0.0113	0.0393	0.198
	FHR	0.9423	0.9085	0.9658	<0.001
	Predicting HFR from prior:				
	HFR	0.9932	0.9901	0.9964	<0.001
	FHR	-0.0066	-0.0112	-0.0027	0.003
	Covariance of HFR and FHR model residuals	0.0001	-0.0009	0.0008	0.826
Male victims:					
	Predicting FHR from prior:				
	HFR	0.0153	-0.0096	0.0355	0.202
	FHR	0.9413	0.9090	0.9654	<0.001
	Predicting HFR from prior:				
	HFR	0.9930	0.9899	0.9964	<0.001
	FHR	-0.0062	-0.0110	-0.0023	0.004
	Covariance of HFR and FHR model residuals	0.0001	-0.0007	0.0008	0.823
Female victims:					
	Predicting FHR from prior:				
	HFR	0.0304	0.0050	0.0549	0.014
	FHR	0.8644	0.8141	0.9008	<0.001
	Predicting HFR from prior:				
	HFR	0.9953	0.9924	0.9982	<0.001
	FHR	-0.0086	-0.0127	-0.0048	<0.001
	Covariance of HFR and FHR model residuals	-0.0004	-0.0018	0.0010	0.540

NOTE: FHR is firearm homicide rate; HFR is household firearm ownership rate. Coefficients for cross-lagged effects (HFR -> FHR; FHR -> HFR) describe the standardized expected differences in the outcome at time 2 for groups with identical outcomes at time 1 but which differ by one standard deviation on the predictor. Coefficients for the association of HFR with later HFR and FHR with later FHR are autocorrelations. The covariance of residuals are the partial correlations between these standardized variables at time *t* conditioned on their *t*-1 values.

eTable 4. Standardized Coefficients for Homicide Rate Models: Subgroup Analyses

			95% confidence interval		
**Analysis**	**Variable**	**Coefficient**	**Lower**	**Upper**	**p**
White Victims:					
	Predicting FHR from prior:				
	HFR	0.0354	0.0054	0.0708	0.054
	FHR	0.8725	0.8405	0.9040	<0.001
	Predicting HFR from prior:				
	HFR	0.9939	0.9913	0.9969	<0.001
	FHR	-0.0062	-0.0107	-0.0032	0.001
	Covariance of HFR and FHR model residuals	-0.0009	-0.0021	0.0002	0.136
Non-white victims					
	Predicting FHR from prior:				
	HFR	0.0092	-0.0070	0.0250	0.253
	FHR	0.9725	0.9555	0.9897	<0.001
	Predicting HFR from prior:				
	HFR	0.9936	0.9902	0.9972	<0.001
	FHR	-0.0037	-0.0086	0.000	0.089
	Covariance of HFR and FHR model residuals	0.0003	-0.0003	0.001	0.299
Urban victims					
	Predicting FHR from prior:				
	HFR	0.0355	-0.0025	0.0834	0.114
	FHR	0.9276	0.8813	0.9606	<0.001
	Predicting HFR from prior:				
	HFR	0.9877	0.9818	0.9948	<0.001
	FHR	-0.0063	-0.0148	0.0004	0.091
	Covariance of HFR and FHR model residuals	0.0009	-0.0007	0.0025	0.245
Non Urban victims					
	Predicting FHR from prior:				
	HFR	0.0136	-0.0058	0.0389	0.220
	FHR	0.9345	0.9096	0.9501	<0.001
	Predicting HFR from prior:				
	HFR	1.0018	0.9969	1.0054	<0.001
	FHR	-0.0107	-0.0156	-0.0062	<0.001
	Covariance of HFR and FHR model residuals	-0.0002	-0.0015	0.0006	0.641

NOTE: FHR is firearm homicide rate; HFR is household firearm ownership rate. Coefficients for cross-lagged effects (HFR -> FHR; FHR -> HFR) describe the standardized expected differences in the outcome at time 2 for groups with identical outcomes at time 1 but which differ by one standard deviation on the predictor. Coefficients for the association of HFR with later HFR and FHR with later FHR are autocorrelations. The covariance of residuals are the partial correlations between these standardized variables at time *t* conditioned on their *t*-1 values.

eTable 5. Standardized Coefficients for Homicide Rate Models: Sensitivity Analyses

© 2024 Morral AR et al. *JAMA Network Open*

Analysis	Variable	Coefficient	95% confidence interval		p
			Lower	Upper	
Full Population: Nonfirearm homicide rate (NFHR)					
	Predicting NFHR from prior:				
	HFR	0.0047	-0.0177	0.0171	0.589
	NFHR	0.8279	0.7813	0.8624	<0.001
	Predicting HFR from prior:				
	HFR	0.9930	0.9896	0.9965	<0.001
	NFHR	-0.0073	-0.0122	-0.0031	<0.001
	Covariance of HFR and NHFR residuals	-0.0005	-0.0013	0.0003	0.208
Full Population: Total homicide rate (THR)					
	Predicting *THR* from prior:				
	HFR	0.0169	-0.0071	0.0366	0.129
	THR	0.9306	0.8985	0.9537	<0.001
	Predicting HFR from prior:				
	HFR	0.9931	0.9899	0.9963	<0.001
	THR	-0.0069	-0.0117	-0.0029	0.002
	Covariance of HFR and THR residuals	0.0001	-0.0009	0.0008	0.841

NOTE: NFHR is non-firearm homicide rate; HFR is household firearm ownership rate. Coefficients for cross-lagged effects (HFR -> NFHR; NFHR -> HFR) describe the expected differences in the standardized outcome at time 2 for groups which differ by one standard deviation on the predictor at time 1. Coefficients for the association of HFR with later HFR and NHR with later NHR are autoregressions. The covariance of residuals are the partial correlations between these standardized variables at time *t* conditioned on their *t*-1 values.

© 2024 Morral AR et al. *JAMA Network Open*
