# Supplementary material for: Household Firearm Ownership and Firearm Mortality
Source: JAMA Netw Open. 2024 Aug 21;7(8):e2429335. doi: 10.1001/jamanetworkopen.2024.29335 (PMC11339659; doi:10.1001/jamanetworkopen.2024.29335)
Supplement: Supplement 2. — Data Sharing Statement [file jamanetwopen-e2429335-s002.pdf]

Data Sharing Statement

Morral. Household Firearm Ownership and Firearm Mortality. *JAMA Netw Open*. Published August 21, 2024. doi:10.1001/jamanetworkopen.2024.29335

Data
Data available: Yes
Data types: Deidentified participant data
How to access data: Mortality data is publicly available through CDC Wonder. Household firearm ownership data was previously published as part of a March 2024 JAMA Network Open paper.
When available: With publication

Supporting Documents
Document types: None

Additional Information
Who can access the data: The data are publicly available.
Types of analyses: For any purpose
Mechanisms of data availability: Data are publicly available.
Any additional restrictions: None
